# Supplementary material for: Maternal asthma imprints fetal lung ILC2s via glucocorticoid signaling leading to worsened allergic airway inflammation in murine adult offspring
Source: Nat Commun. 2025 Jan 13;16:631. doi: 10.1038/s41467-025-55941-8 (PMC11730321; doi:10.1038/s41467-025-55941-8)
Supplement: Supplementary file 1 — Supplementary Information [file 41467_2025_55941_MOESM1_ESM.pdf]

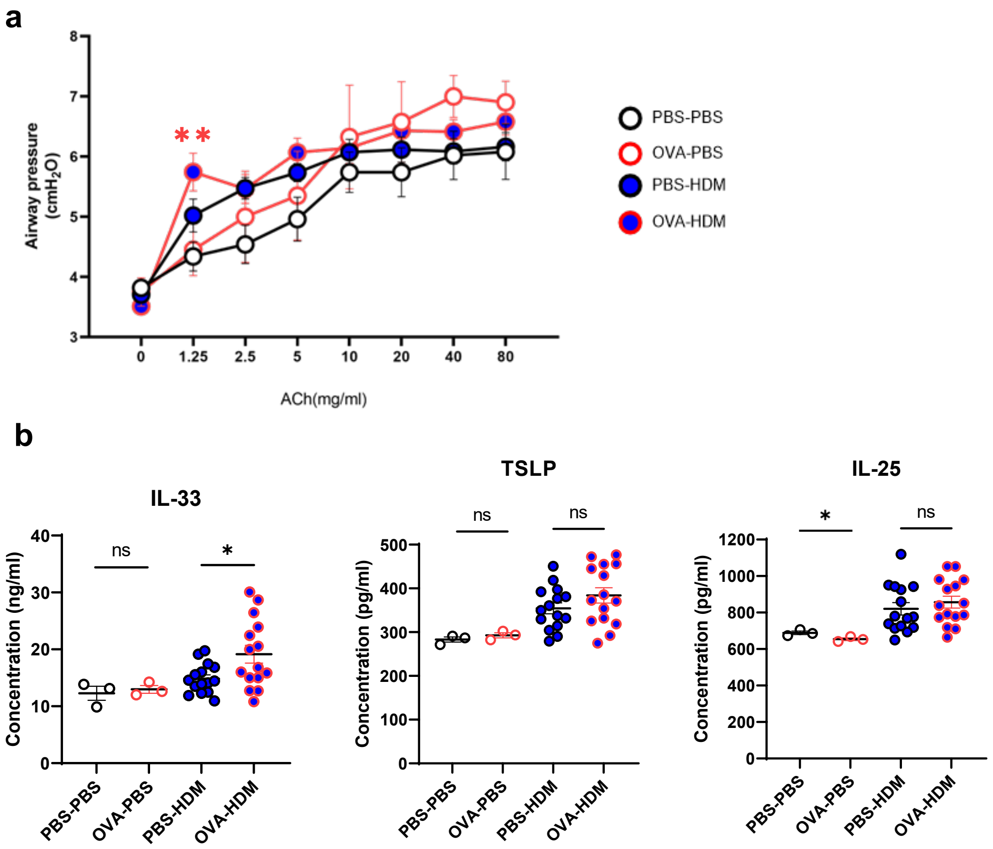

Supplementary Fig. 1: Airway hyperresponsiveness and levels of epithelial-derived cytokines in adult offspring from OVA asthmatic or control mothers

a, Assessment of acetylcholine-induced airway hyperresponsiveness in adult offspring of asthmatic or control mothers, with absolute airway pressure values shown on the Y-axis. **b,** Levels of IL-33, TSLP, and IL-25 in the lungs of adult offspring from OVA-asthmatic or control mothers. Cytokine levels were measured in lung homogenate supernatants by ELISA. In **a** and **b**, data were pooled from four independent experiments **a**, each experiment with two pregnant dams, or two independent experiments **b**, each experiment with two pregnant dams. Sample sizes were as follows: **a** PBS-PBS: n = 5, OVA-PBS: n = 4, PBS-HDM: n = 15, OVA-HDM: n = 16; **b** PBS-PBS: n = 3, OVA-PBS: n = 3, PBS-HDM: n = 15, OVA-HDM: n = 15. In **a, b,** data are presented as the mean ± SEM. In **b**, each dot represents an individual mouse. Statistical analyses were performed two-way ANOVA followed by Tukey's test in **a** and unpaired two-tailed Student's t-test in **b**. *p < 0.05; **p < 0.01; ns, not significant.

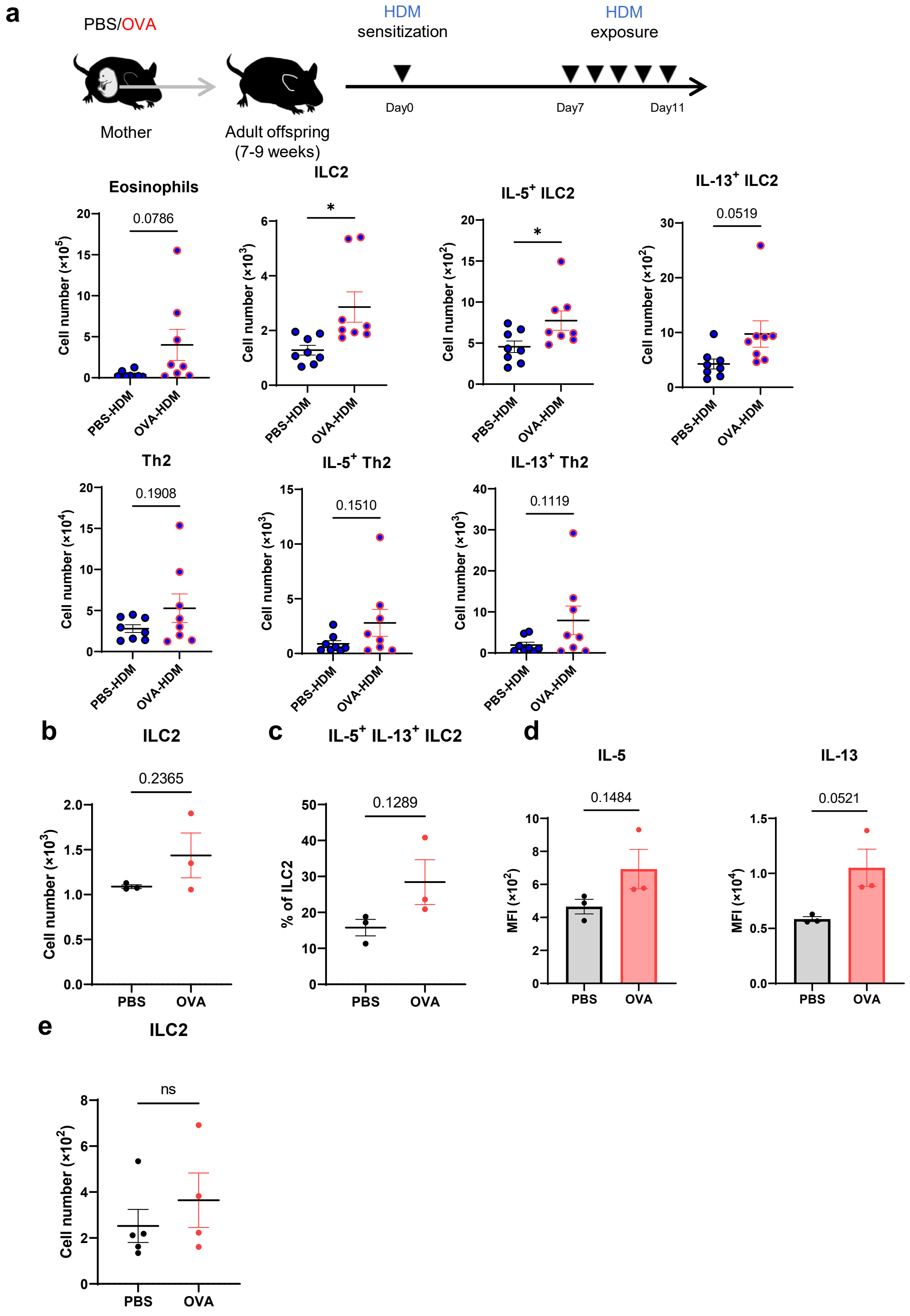

Supplementary Fig. 2: Lung ILC2s in adult offspring of asthmatic mothers are hyperactive.

a, Model of adult offspring sensitized and exposed to HDM from asthmatic or control mothers. Changes in eosinophil counts, ILC2 numbers, Th2 cell counts, as well as the numbers of cytokine-producing ILC2s and Th2 cells in the lungs of adult offspring are presented. **b, c,** Post-culture cell numbers and cytokine production of lung ILC2s sorted from adult offspring of asthmatic or control mothers were analyzed by flow cytometry. **d**, median fluorescence intensity (MFI) of IL-5 and IL-13 in cultured lung ILC2s. **e**, Counts of lung ILC2s in recipient mice transplanted with lung ILC2s sorted from adult offspring derived from asthmatic or control mothers. In **a - e**, data were from one experiment **a** with two or three pregnant dams per group, or representative of two independent experiments **b - d**, each experiment with one pregnant dam per group, or one experiment **e** with one pregnant dam per group. Sample sizes were as follows: **a** PBS-HDM: n = 8, OVA-HDM: n = 8; **b - d** PBS: n = 3, OVA: n = 3; **e** PBS: n = 5, OVA: n = 4. In **a - e**, data are presented as the mean ± SEM. In **a, e**, each dot represents an individual mouse. In **b - d**, each dot represents an individual well from an in vitro experiment, with cells sorted and pooled from PBS (n = 4) and OVA (n = 4) groups of mice. Statistical analyses were performed unpaired two-tailed Student's *t*-test in **e**. *$p < 0.05$; ns, not significant.

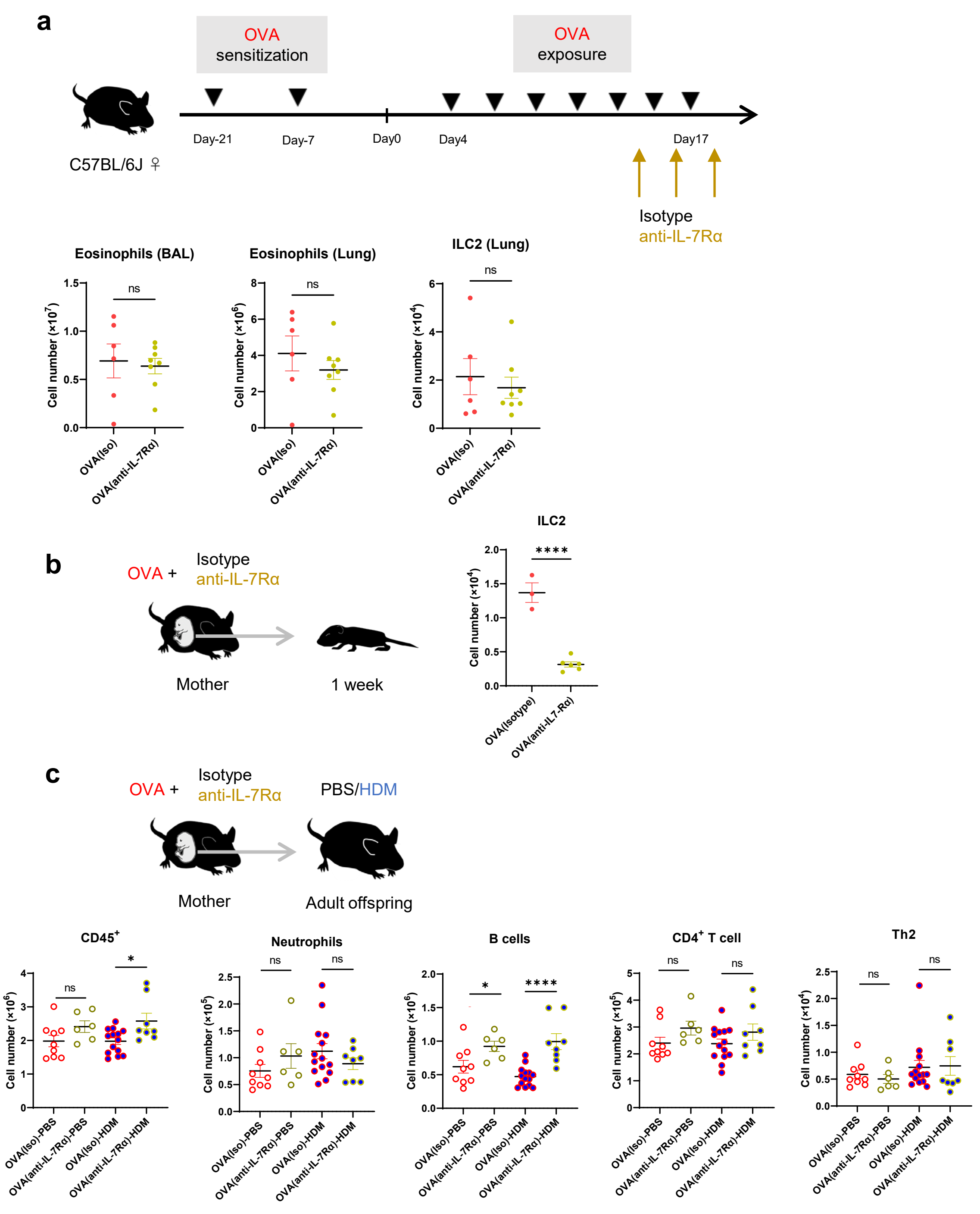

Supplementary Fig. 3: Maternal anti-IL-7Ra antibody administration alters offspring lung immune cells. a, Asthmatic and antibody-treatment protocol in Fig. 3b was implemented in non-pregnant WT mice; counts of eosinophils and ILC2s in BAL and lung in the isotype and anti-IL-7Ra antibody treatment groups. **b,** Asthmatic mothers were treated with isotype control or anti-IL-7Ra antibodies, and their offspring were evaluated at 1 week of age. Changes in the number of ILC2s in the lungs of the offspring. **c,** Changes in the number of cells of each immune cell type in the lungs of adult offspring of asthmatic mothers treated with antibodies. In **a - c**, data were pooled from two independent experiments **a, c**, each experiment with one or two pregnant dams c, or one experiment **b** with one pregnant dam per group. Sample sizes were as follows: **a** OVA (Iso): n = 6, OVA (anti-IL-7Rα): n = 8; **b** OVA (Isotype): n = 3, OVA (anti-IL-7Rα): n = 6; **c** OVA (Iso)-PBS: n = 9, OVA (anti-IL-7Rα)-PBS: n = 6, OVA (Iso)-HDM: n = 14, OVA (anti-IL-7Rα)-HDM: n = 8. In **a - c**, data are presented as the mean ± SEM. Each dot represents an individual mouse. Statistical analyses were performed unpaired two-tailed Student's *t*-test. *$p < 0.05$; ****$p < 0.0001$; ns, not significant.

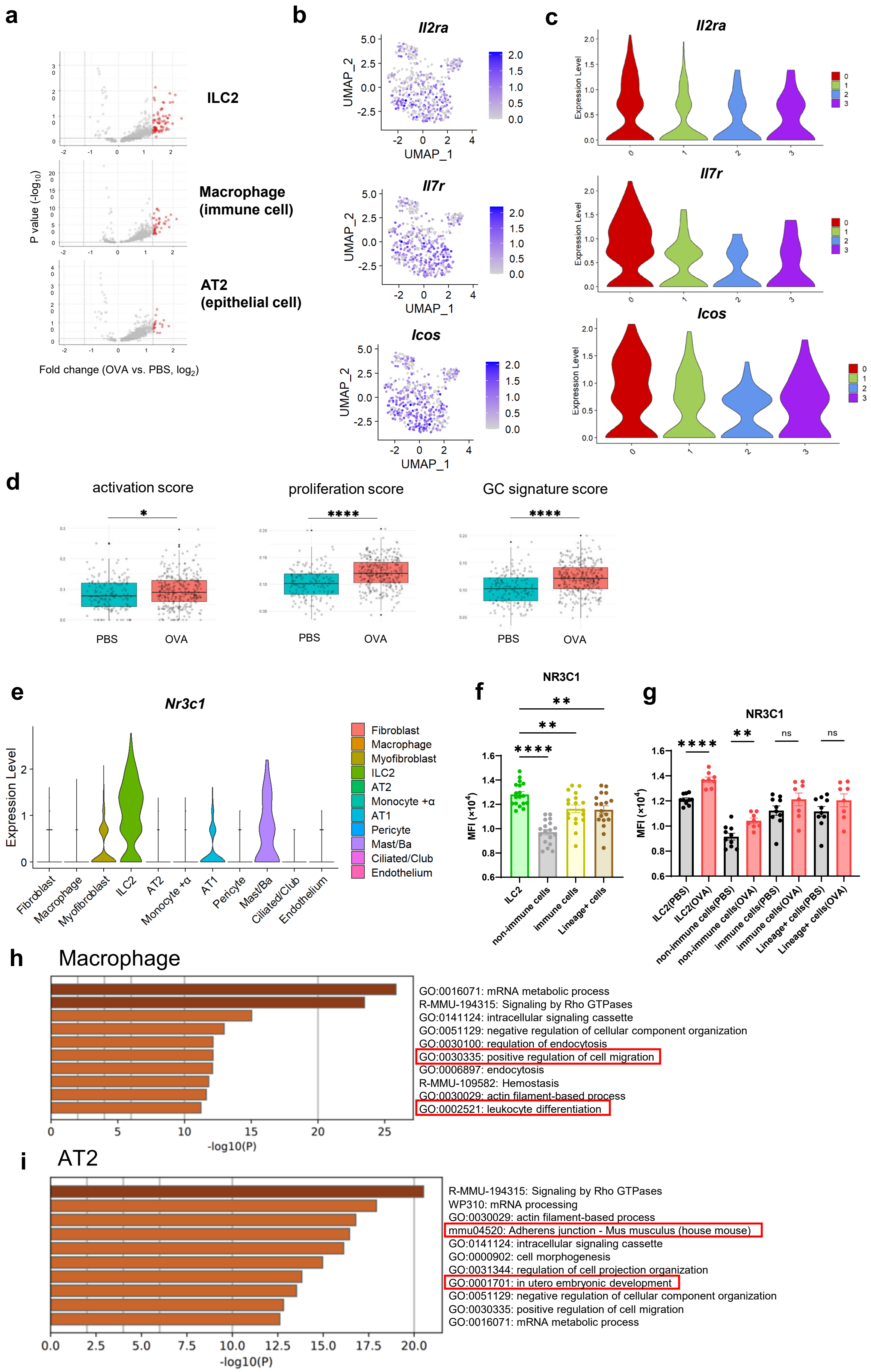
a
ILC2
Macrophage (immune cell)
AT2 (epithelial cell)
P value (-log10)
Fold change (OVA vs. PBS, log2)
b
Il2ra
Il7r
Icos
UMAP_1
UMAP_2
c
Il2ra
Il7r
Icos
Expression Level
d
activation score
proliferation score
GC signature score
PBS
OVA
e
Nr3c1
Expression Level
Fibroblast
Macrophage
Myofibroblast
ILC2
AT2
Monocyte +α
AT1
Pericyte
Mast/Ba
Ciliated/Club
Endothelium
f
NR3C1
MFI (×10⁴)
ILC2
non-immune cells
immune cells
Lineage+ cells
g
NR3C1
MFI (×10⁴)
ILC2(PBS)
ILC2(OVA)
non-immune cells(PBS)
non-immune cells(OVA)
immune cells(PBS)
immune cells(OVA)
Lineage+ cells(PBS)
Lineage+ cells(OVA)
ns
h
Macrophage
GO:0016071: mRNA metabolic process
R-MMU-194315: Signaling by Rho GTPases
GO:0141124: intracellular signaling cassette
GO:0051129: negative regulation of cellular component organization
GO:0030100: regulation of endocytosis
GO:0030335: positive regulation of cell migration
GO:0006897: endocytosis
R-MMU-109582: Hemostasis
GO:0030029: actin filament-based process
GO:0002521: leukocyte differentiation
-log10(P)
i
AT2
R-MMU-194315: Signaling by Rho GTPases
WP310: mRNA processing
GO:0030029: actin filament-based process
mmu04520: Adherens junction - Mus musculus (house mouse)
GO:0141124: intracellular signaling cassette
GO:0000902: cell morphogenesis
GO:0031344: regulation of cell projection organization
GO:0001701: in utero embryonic development
GO:0051129: negative regulation of cellular component organization
GO:0030335: positive regulation of cell migration
GO:0016071: mRNA metabolic process
-log10(P)

Supplementary Fig. 4: Fetal lung ILC2 proportions are altered by maternal asthma during pregnancy.
a, Volcano plot illustrating group differences for each representative cell type in the fetal lung. **b, c,** Relative expression of representative genes, including cell surface markers, in ILC2s (**b**), and a violin plot displaying expression patterns among ILC2 subclusters (**c**). **d,** Box and dot plot demonstrating the characteristic activity of each cell, scored and compared between the groups. **e,** Violin plot illustrating the expression pattern of *Nr3C1* for each cell type in the fetal lung. **f, g,** MFI of Nr3C1 in flow cytometry for each cell type in the fetal lung (**f**), and comparison between the groups for each cell type (**g**). **h, i,** Enrichment analysis of upregulated genes in macrophages (**h**) and AT2 (**i**) of fetal lungs from asthmatic mothers compared to controls (top 10 positions shown). In **a - i**, data were derived from one experiment **a – e, h, j,** with one pregnant dam per group, or two independent experiments **f, g,** each experiment with one pregnant dam per group. Sample sizes were as follows: **f** ILC2, non-immune cells, immune cells, and lineage+ cells: n = 18 for each group; **g** ILC2 (PBS: n = 10, OVA: n = 8), non-immune cells (PBS: n = 10, OVA: n = 8), immune cells (PBS: n = 10, OVA: n = 8), and lineage+ cells (PBS: n = 10, OVA: n = 8). In **f, g,** data are presented as the mean ± SEM. In **f, g,** each dot represents an individual mouse. For box plots in **d**: Data are displayed as min to max, showing all points. The midline represents the median, the box indicates the interquartile range (IQR) between the 25th (Q1) and 75th (Q3) percentiles, and the whiskers extend to the minimum and maximum values within 1.5 times the IQR. Statistical analyses were performed two-tailed Wilcoxon rank sum test in **d**, one-way ANOVA followed by Dunnett's test in **f** and unpaired two-tailed Student's *t*-test in **g**. *p < 0.05; **p < 0.01; ****p < 0.0001; ns, not significant.

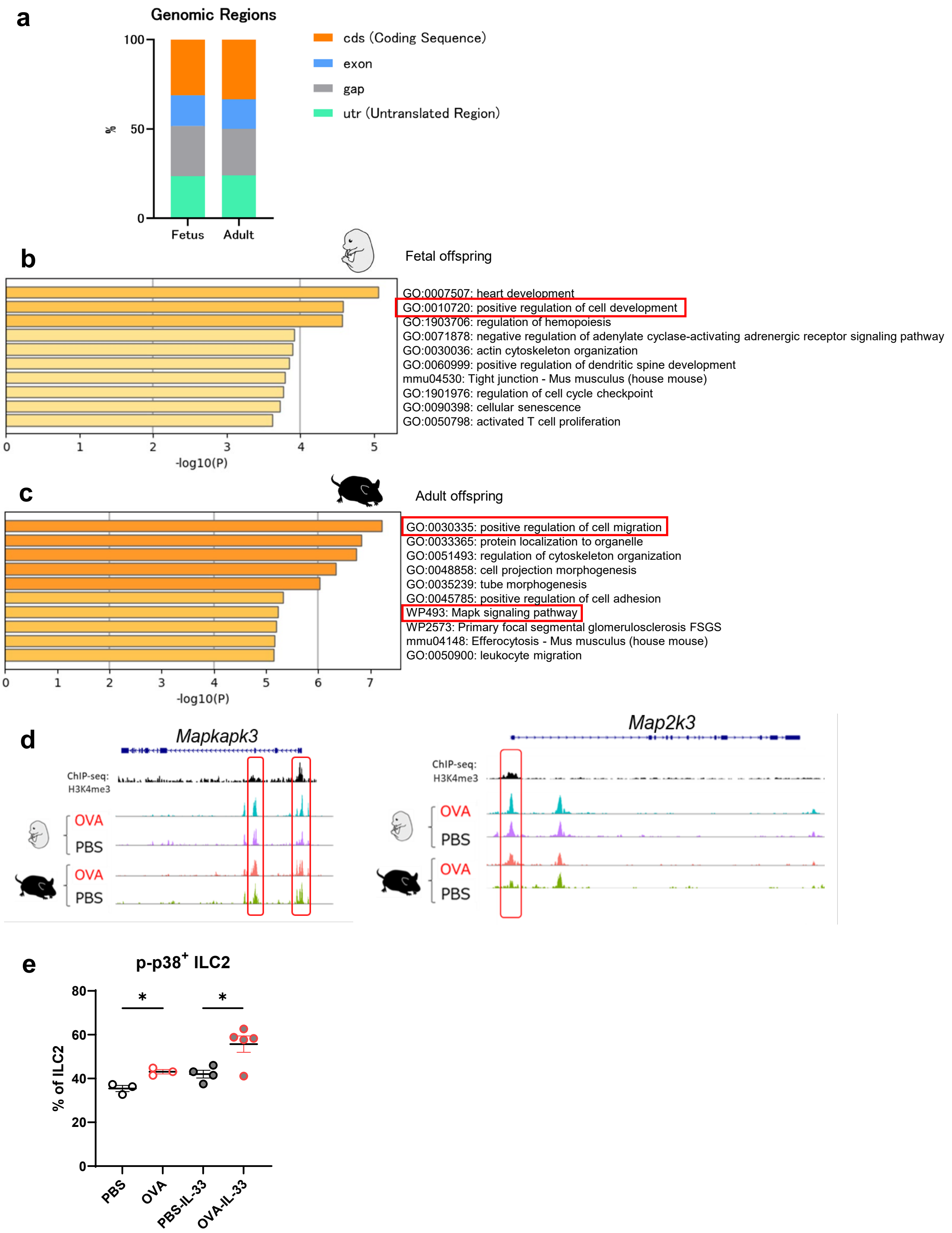

Supplementary Fig. 5: MapK signaling is activated in lung ILC2s in fetal and adult offspring of asthmatic mothers.

a, The proportion of genomic region types in the differentially accessible sites in fetuses and adult offspring of asthmatic mothers. **b,** Enrichment analysis of genes associated with regions that were more open in fetal lung ILC2s from asthmatic mothers (top 10 positions shown). **c,** Enrichment analysis of genes associated with regions that were more open in adult offspring lung ILC2s from asthmatic mothers (top 10 positions shown). **d,** Results of scATAC-seq and ChIP-seq in lung ILC2s of offspring from asthmatic or control mothers in gene regions associated with the MAPK pathway. **e,** Percentage of lung ILC2s expressing phosphorylated p38 in adult offspring from asthmatic or control mothers. The left two groups represent untreated offspring, while the right two groups received intranasal IL-33 (0.25 μg) treatment for two days *in vivo*. In **a - e**, data were derived from one experiment **a - d** with one pregnant dam per group, or one experiment **e** with two pregnant dams per group. Sample sizes were as follows: **e** PBS: n = 3, OVA: n = 3, PBS-IL-33: n = 4, OVA-IL-33: n = 5. In **e,** data are presented as the mean ± SEM. In **e**, each dot represents an individual mouse. Statistical analyses were performed unpaired two-tailed Student's *t*-test. *$p < 0.05$.

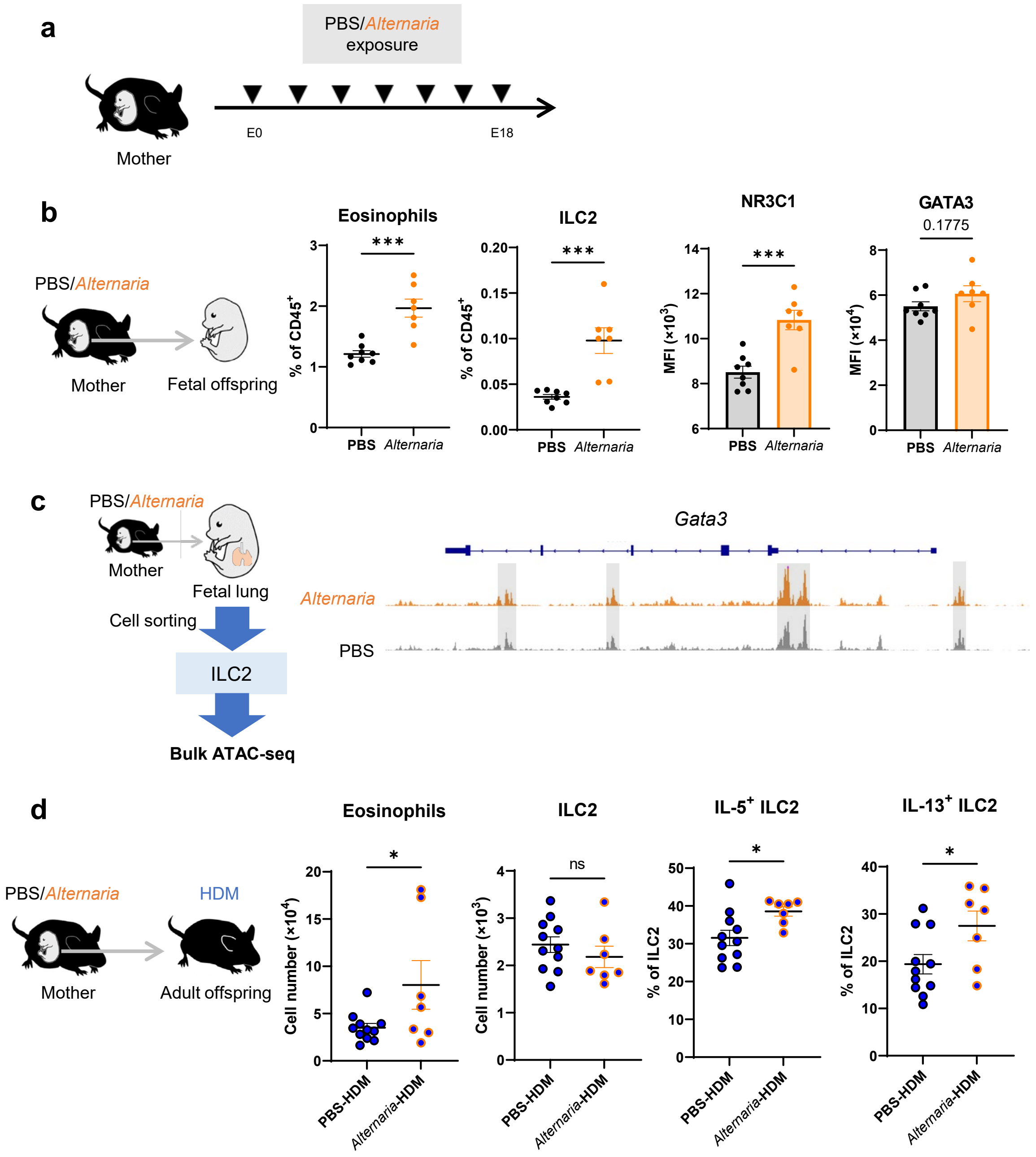

Supplementary Fig. 6: *Alternaria*-induced asthma during pregnancy alters lung ILC2s in offspring.
a, Model of chronic maternal asthma during pregnancy with *Alternaria*: Following mating, pregnant females were treated intranasally with either PBS or *Alternaria* every three days from gestation day 0 to day 18. **b,** Percentage of eosinophils and ILC2s in the lungs of fetuses from *Alternaria* asthmatic or control mothers (day 18 of gestation), with MFI of NR3C1 and GATA3 in fetal lung ILC2s shown as bar plots. **c,** Experimental design of bulkATAC-seq on ILC2s sorted from fetal lungs of mothers with *Alternaria*-induced asthma or controls. Shown are bulkATAC-seq results at the *Gata3* locus in fetal lung ILC2s. **d,** Changes in eosinophil and ILC2 counts, including cytokine-producing ILC2s, in the lungs of adult offspring from *Alternaria* asthmatic or control mothers. ILC2s were stimulated with PMA/ionomycin for 4 hours, and cytokine production was assessed by intracellular staining. In **b - d**, data were from one experiment **b**, with one pregnant dam per group, or one experiment **c**, with one or two pregnant dams per group, or representative of two independent experiments **d**, each experiment with two pregnant dams per group. Sample sizes were as follows: **b** PBS: n = 8, *Alternaria*: n = 7; **d** PBS-HDM: n = 11, *Alternaria*-HDM: n = 7. In **b, d,** data are presented as the mean ± SEM. In **b, d,** each dot represents an individual mouse. Statistical analyses were performed unpaired two-tailed Student's t-test in **b, d.** *$p < 0.05$; ****$p < 0.0001$; ns, not significant.

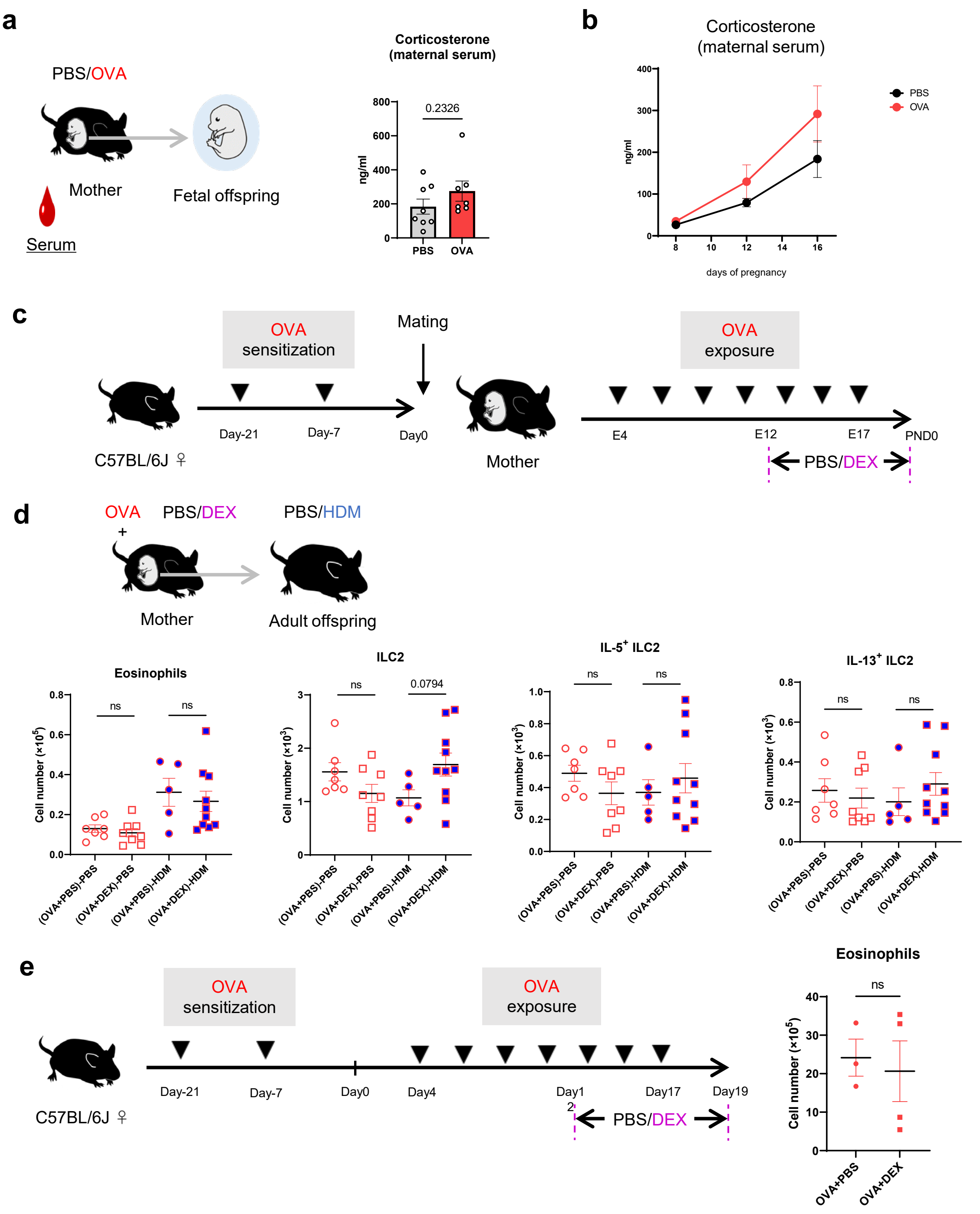

Supplementary Fig. 7: Asthma during pregnancy raises maternal corticosterone levels and may impact lung ILC2s in offspring

a, b, Serum corticosterone concentration in asthmatic or control mothers (**a**) and its time course (**b**). **c,** Model of chronic asthma induction and glucocorticoid treatment during pregnancy. Female mice developed asthma during pregnancy (Fig. 1a) and were also treated orally with water containing PBS or DEX from day 12 of gestation until the end of pregnancy (Fig. 6b). **d,** Adult offspring of PBS- or DEX-treated asthmatic mothers received PBS or HDM intranasally (Fig. 1a). Changes in eosinophil and ILC2 cell numbers in the lungs of adult offspring; ILC2s were stimulated with PMA/ionomycin for 4 h, and cytokine production was assessed by intracellular staining. **e,** Using non-pregnant WT mice to assess the effects of PBS or DEX treatment on the OVA asthma model. The figure shows the eosinophil counts in the lungs. In **a, b, d, e**, data were pooled from three independent experiments **a, b,** with one pregnant dam per group, or one experiment **d,** with two or three pregnant dams per group, or one experiment **e**. Sample sizes were as follows: **a** PBS: n = 8, OVA: n = 7; **b** PBS: n = 8, OVA: n = 6; **d** (OVA+PBS)-PBS: n = 7, (OVA+DEX)-PBS: n = 8, (OVA+PBS)-HDM: n = 5, (OVA+DEX)-HDM: n = 10; **e** OVA+PBS: n = 3, OVA+DEX: n = 4. In **a, b, d, e,** data are presented as the mean ± SEM. In **a, d, e**, each dot represents an individual mouse. Statistical analyses were performed unpaired two-tailed Student's *t*-test in **a, d, e**. ns, not significant.

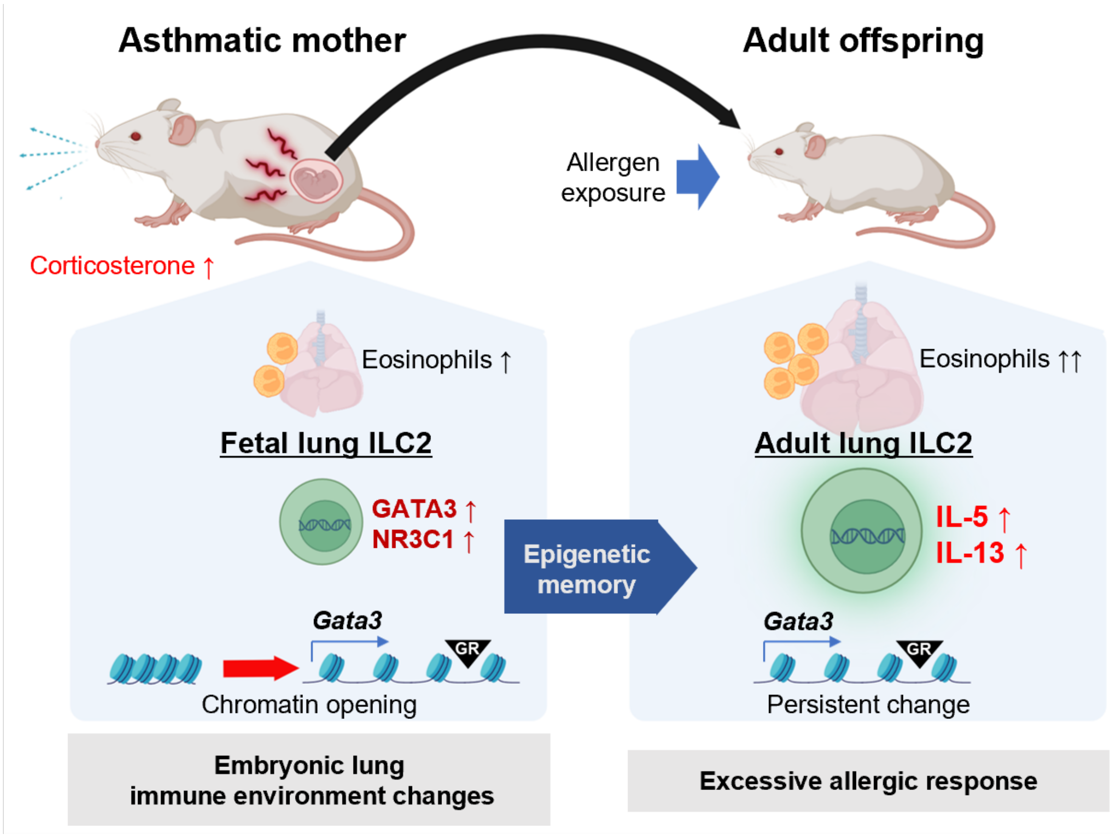

Supplementary Fig. 8: Schematic model showing that prenatal maternal asthma imprints fetal lung ILC2s via glucocorticoid signaling and enhances their function in adulthood.
Maternal asthma has long-term effects that alter fetal lung ILC2s and lead to increased lung ILC2 responsiveness in adults and that the mechanism involves epigenetic changes and glucocorticoid signaling. Created in BioRender. Lab, I. (2024) https://BioRender.com/w94z021

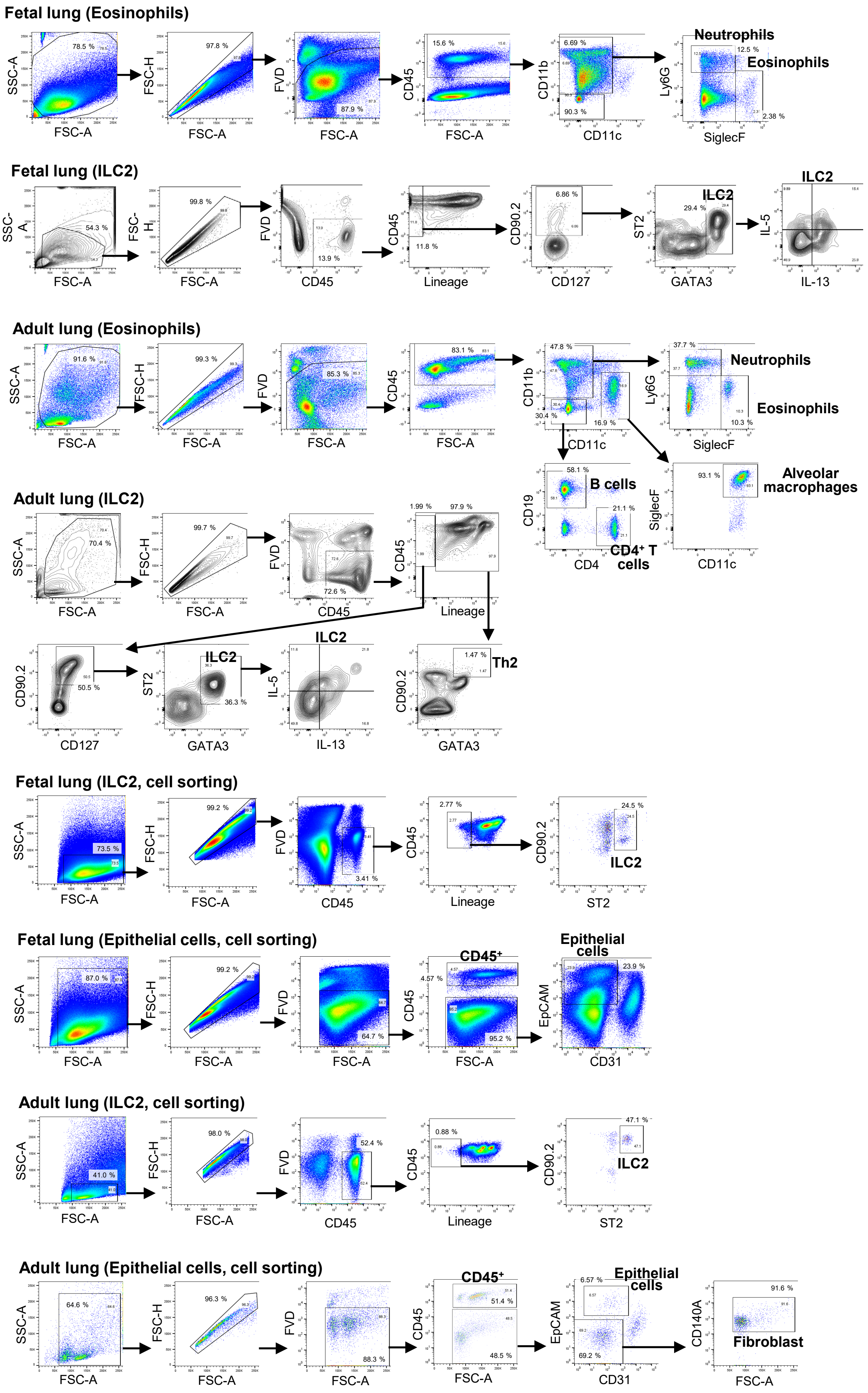

Supplementary Fig. 9: Gating strategy for all experiments.
