# Supplementary material for: Maternal asthma imprints fetal lung ILC2s via glucocorticoid signaling leading to worsened allergic airway inflammation in murine adult offspring
Source: Nat Commun. 2025 Jan 13;16:631. doi: 10.1038/s41467-025-55941-8 (PMC11730321; doi:10.1038/s41467-025-55941-8)
Supplement: Supplementary file 2 — Description of Additional Supplementary Files [file 41467_2025_55941_MOESM2_ESM.pdf]

Description of Additional Supplementary Files

Supplementary Data 1: Absolute airway pressure values measured during airway hyperresponsiveness tests in adult offspring from OVA-asthmatic or control mothers.

Supplementary Data 2: List of differentially expressed genes (DEGs) in ILC2s, macrophages, and AT2 in fetal lungs from OVA-asthmatic or control mothers. DEGs between OVA and PBS were identified using the FindMarkers function from the Seurat package. Statistical significance was determined using the Mann-Whitney U test (two-sided), with Benjamini-Hochberg correction applied for multiple comparisons.

Supplementary Data 3: Gene sets used to score ILC2s in fetal lungs from OVA-asthmatic or control mothers.

Supplementary Data 4: List of antibodies used in the experiments.
