# Supplementary material for: Maternal asthma imprints fetal lung ILC2s via glucocorticoid signaling leading to worsened allergic airway inflammation in murine adult offspring
Source: Nat Commun. 2025 Jan 13;16:631. doi: 10.1038/s41467-025-55941-8 (PMC11730321; doi:10.1038/s41467-025-55941-8)
Supplement: Supplementary file 7 — Reporting Summary [file 41467_2025_55941_MOESM7_ESM.pdf]

nature portfolio

Corresponding author(s): Minako Ito

Last updated by author(s): Oct 31, 2024

Reporting Summary

Nature Portfolio wishes to improve the reproducibility of the work that we publish. This form provides structure for consistency and transparency in reporting. For further information on Nature Portfolio policies, see our Editorial Policies and the Editorial Policy Checklist.

Statistics

For all statistical analyses, confirm that the following items are present in the figure legend, table legend, main text, or Methods section.

n/a	Confirmed	
☐	☒	The exact sample size (*n*) for each experimental group/condition, given as a discrete number and unit of measurement
☐	☒	A statement on whether measurements were taken from distinct samples or whether the same sample was measured repeatedly
☐	☒	The statistical test(s) used AND whether they are one- or two-sided<br>*Only common tests should be described solely by name; describe more complex techniques in the Methods section.*
☒	☐	A description of all covariates tested
☐	☒	A description of any assumptions or corrections, such as tests of normality and adjustment for multiple comparisons
☐	☒	A full description of the statistical parameters including central tendency (e.g. means) or other basic estimates (e.g. regression coefficient) AND variation (e.g. standard deviation) or associated estimates of uncertainty (e.g. confidence intervals)
☐	☒	For null hypothesis testing, the test statistic (e.g. *F*, *t*, *r*) with confidence intervals, effect sizes, degrees of freedom and *P* value noted<br>*Give P values as exact values whenever suitable.*
☒	☐	For Bayesian analysis, information on the choice of priors and Markov chain Monte Carlo settings
☒	☐	For hierarchical and complex designs, identification of the appropriate level for tests and full reporting of outcomes
☒	☐	Estimates of effect sizes (e.g. Cohen's *d*, Pearson's *r*), indicating how they were calculated

Our web collection on statistics for biologists contains articles on many of the points above.

Software and code

Policy information about availability of computer code

Data collection

Flow cytometry analysis: Data were performed by LSRFortessa (BD Biosciences) or FACSMelody (BD Biosciences).

Single-cell RNA sequencing:
Libraries were prepared according to the protocol of Chromuim Next GEM Single Cell 5' Reagent Kits v2 (Dual Index) (10x Genomics, PN-1000263) using a Chromium controller (10x Genomics). Sequencing was performed by Novaseq 6000 (Illumina).

Single-nucleus RNA and ATAC sequencing:
The nuclei were isolated according to the protocol of “Nuclei Isolation from Embroynic Mouse Brain for Single Cell Multiome ATAC + Gene Expression Sequencing, Protocol 1” (10x Genomics, CG000366). Libraries were prepared according to the protocol of Chromuim Next GEM Single Cell Multiome ATAC + Gene Expression Kit using a Chromium controller (10x Genomics). Sequencing was performed by Novaseq 6000 (Illumina).

bulkATAC sequencing:
Libraries were prepared according to the protocol of ATAC-Seq Kit (Active Motif, 53150). Sequencing was performed by Novaseq 6000 (Illumina).

H3K4me3 Carrier Assisted ChIP-seq (CATCH-seq):
Adaptor ligation was performed by NEBNext Ultra II DNA Library Prep Kit for Illumina (E7645, NEB) , and the libraries were purified by 1.8x SPRIselect beads (B23318, Beckman Coulter). The libraries were sequenced on Novaseq 6000 (Illumina) with paired-end reads.

Data analysis	Flow cytometry analysis: Data were analyzed with FlowJo (version 10, BD Biosciences).<br><br>Single-cell RNA sequencing:<br>From the NGS read data, FASTQ files integrated with the Totalseq Hashtags were generated by Cell Ranger Multi function (v7, 10x Genomics). Further analyses were performed using Seurat (version 5.0.1) in the RStudio platform (Version R 4.3.0).<br><br>Single-nucleus RNA and ATAC sequencing:<br>From the NGS read data, FASTQ files were generated by CellRanger Arc mkfastq, and then integrated with ATAC and Gene Expression the were generated by Cell Ranger Arc (v2.0.1, 10x Genomics). Further analysis was performed using Seurat (Version 5.0.1) and Signac (1.12.0) on the RStudio platform (Version R 4.3.0).<br><br>bulkATAC sequencing:<br>All sequencing reads after trimming by TrimGalore (version 0.6.10) and Cutadapt (version 4.4) were aligned to the mouse and genome sequences (mouse genome; Genome Reference Consortium Mouse Build 38, mm10, Ensembl v.98) using Bowtie 2 (version 2.5.2). SAMtools (version 1.18) was used to manipulate alignments in the SAM and BAM formats. For visualization of ATAC-seq using the Integrative Genomics Viewer (version 2.17.0), genome coverage tracks were generated using bamCoverage from deepTools (version 3.5.4) with the parameters '--binSize 10 --normalizeUsing RPGC'.<br><br>CATCH-seq:<br>All sequencing reads after trimming by TrimGalore (version 0.6.10) and Cutadapt (version 4.4) were aligned to the mouse and genome sequences (mouse genome; Genome Reference Consortium Mouse Build 38, mm10, Ensembl v.98) using Bowtie 2 (version 2.5.2) with the '-q -N 1 -L 25 --no-mixed --no-discordant' options. After the removal of PCR duplicates using Sambamba (version 1.0.0) with the parameters 'markdup -r -t 2'. SAMtools (version 1.18) was used to manipulate alignments in the SAM and BAM formats. For visualization of ChIP-seq using the Integrative Genomics Viewer (version 2.17.0), genome coverage tracks were generated using bamCoverage from deepTools (version 3.5.4) with the parameters '--scaleFactor Scaling_factor --binSize 50 --normalizeUsing RPKM'.<br><br>Statistical analyses were performed using GraphPad Prism (Version 9).

For manuscripts utilizing custom algorithms or software that are central to the research but not yet described in published literature, software must be made available to editors and reviewers. We strongly encourage code deposition in a community repository (e.g. GitHub). See the Nature Portfolio guidelines for submitting code & software for further information.

Data

Policy information about availability of data

All manuscripts must include a data availability statement. This statement should provide the following information, where applicable:

- Accession codes, unique identifiers, or web links for publicly available datasets
- A description of any restrictions on data availability
- For clinical datasets or third party data, please ensure that the statement adheres to our policy

Single cell RNA-seq data for fetal lung ILC2 from DEX- or PBS-treated mothers (GSE262718), single nucleus RNA-seq (GSE262715), ATAC-seq (GSE262716) and ChIP-seq (GSE262949) data for lung cells from OVA- or PBS-treated mothers are available.

Research involving human participants, their data, or biological material

Policy information about studies with human participants or human data. See also policy information about sex, gender (identity/presentation), and sexual orientation and race, ethnicity and racism.

Reporting on sex and gender	Not applicable
Reporting on race, ethnicity, or other socially relevant groupings	Not applicable
Population characteristics	Not applicable
Recruitment	Not applicable
Ethics oversight	Not applicable

Note that full information on the approval of the study protocol must also be provided in the manuscript.

Field-specific reporting

Please select the one below that is the best fit for your research. If you are not sure, read the appropriate sections before making your selection.

- [x] Life sciences
- [ ] Behavioural & social sciences
- [ ] Ecological, evolutionary & environmental sciences

For a reference copy of the document with all sections, see nature.com/documents/nr-reporting-summary-flat.pdf

Life sciences study design

All studies must disclose on these points even when the disclosure is negative.

Sample size	No statistical methods were used to determine sample size. The sample size for the animal experiments was determined based on preliminary studies and previous experimental experiences.
Data exclusions	In the ILC2 transfer experiments, samples in which ILC2 was not identified in the recipient mouse lung were excluded.
Replication	The number of times each experiment was conducted and the statistical analyses used are detailed in the figure legends.
Randomization	All samples were randomly allocated into experimental groups.
Blinding	The investigators were not blinded to group allocation during data collection and analysis. Lung histology scoring was performed in a blinded manner, with the mean score per mouse calculated.

Reporting for specific materials, systems and methods

We require information from authors about some types of materials, experimental systems and methods used in many studies. Here, indicate whether each material, system or method listed is relevant to your study. If you are not sure if a list item applies to your research, read the appropriate section before selecting a response.

Materials & experimental systems

n/a	Involved in the study	
☐	☒	Antibodies
☒	☐	Eukaryotic cell lines
☒	☐	Palaeontology and archaeology
☐	☒	Animals and other organisms
☒	☐	Clinical data
☒	☐	Dual use research of concern
☒	☐	Plants

Methods

n/a	Involved in the study	
☐	☒	ChIP-seq
☐	☒	Flow cytometry
☒	☐	MRI-based neuroimaging

Antibodies

Antibodies used

[Flow cytometry]
BV650 anti-mouse CD117(c-kit) (Clone 2B8) BioLegend 105853
PerCP anti-mouse CD11b (Clone M1/70) eBioscience 45-0112-82
Biotin anti-mouse CD11b (Clone M1/70) eBioscience 36-0112-85
FITC anti-mouse CD11b (Clone M1/70) eBioscience 11-0112-82
APC anti-mouse CD11c (Clone N418) eBioscience 17-0114-82
Biotin anti-mouse CD11c (Clone HL3) BD Bioscience 553800
FITC anti-mouse CD11c (Clone N418) eBioscience 11-0114-85
BV786 anti-mouse CD11c (Clone HL3) BD Biosciences 563735
BV711 anti-mouse CD127(IL-7R) (Clone SB/199) BD Biosciences 565490
PE-Cy7 anti-mouse CD140a (PDGFRa) (Clone APA5) BioLegend 135912
PE anti-mouse CD170 (SiglecF) (Clone 1RNM44N) eBioscience 12-1702-82
Biotin anti-mouse CD19 (Clone 1D3) eBioscience 13-0193-85
FITC anti-mouse CD19 (Clone 1D3) eBioscience 11-0193-82
BUV737 anti-mouse CD19 (Clone 1D3) BD Biosciences 612781
FITC anti-mouse CD25 (Clone PC61.5) eBioscience 11-0251-82
BV605 anti-mouse CD279 (PD-1) (Clone 29F.1A12) BioLegend 135220
APC anti-mouse CD31 (Clone MEC13.3) BioLegend 102509
PE anti-mouse CD326 (Clone EpCAM) eBioscience 12-5791-83
BV711 anti-mouse CD366 (Clone Tim3) BioLegend 119727
Biotin anti-mouse CD3e (Clone 145-2C11) eBioscience 13-0031-85
FITC anti-mouse CD3e (Clone 145-2C11) eBioscience 11-0031-86
Biotin anti-mouse CD4 (Clone RM4-5) eBioscience 13-0042-85
FITC anti-mouse CD4 (Clone RM4-5) eBioscience 11-0042-85
PE-Cy7 anti-mouse CD4 (Clone RM4-5) eBioscience 25-0042-82
FITC anti-mouse CD44 (Clone IM7) eBioscience 11-0441-81
FITC anti-mouse CD45 (Clone 30-F11) eBioscience 11-0451-85
BV786 anti-mouse CD45 (Clone 30-F11) BD Biosciences 564225
PE anti-mouse CD45.2 (Clone 104) eBioscience 12-0454-83
Biotin anti-mouse CD8A (Clone 53-6.7) BD Bioscience 553029

FITC anti-mouse CD8A (Clone 53-6.7) eBioscience 11-0081-82
BV785 anti-mouse CD8A (Clone 53-6.7) BioLegend 100750
BV510 anti-mouse CD90.2(Thy1.2) (Clone 53-2.1) BioLegend 140319
PE anti-mouse EPCAM (CD326) (Clone G8.8) eBioscience 12-5791-83
PE-Cy7 anti-mouse Gata3 (Clone TWAJ) eBioscience 25-9966-42
PE anti-mouse IL-13 (Clone eBio 13A) eBioscience 12-7133-82
APC anti-mouse IL-5 (Clone TRFK5) BioLegend 504306
APC anti-mouse NR3C1 (Clone BuGR2) eBioscience 17-6189-82
BV605 anti-mouse KLRG1 (Clone 2F1/KLRG1) BioLegend 138419
PE-Cy7 anti-mouse Ly6G (Clone 1A8) eBioscience 25-9668-82
BUV661 anti-mouse Ly6G (Clone 1A8) BD Biosciences 741587
Biotin anti-mouse NK1.1 (Clone PK136) eBioscience 13-5941-85
BUV737 Streptavidin BD Bioscience 612775
PE-Cy7 Streptavidin eBioscience 25-4317-82
BV421 anti-mouse SiglecF (Clone E50-2440) BD Biosciences 562681
BV421 anti-mouse ST2 (Clone U29-93) BD Biosciences 566309
Biotin anti-mouse ST2 (Clone DJ8) mdbioproducts 101001B
BV650 anti-mouse TCRβ (Clone H57-597) BD Biosciences 742483
Biotin anti-mouse TCRγδ (Clone GL3) BD Bioscience 553176
Biotin anti-mouse TER119 (Clone TER-119) BD Bioscience 553672
FITC anti-mouse TER119 (Clone TER-119) eBioscience 11-5921-85
PE anti-mouse Tox (Clone TXRX10) eBioscience 12-6502-82
Fixable Viability Dye eFluor™ 780 eBioscience 65-0865-14
AlexaFluor647-conjugated anti-p-p38 BD Biosciences 612595

[Antibody administration experiments]
InVivoMAb anti-mouse IL-7Rα (CD127) (A7R34) Bio X Cell BE0065
InVivoPlus anti-mouse CD8α (2.43) Bio X Cell BP0061
InVivoPlus anti-mouse CD4 (GK1.5) Bio X Cell BP0003-1

[Single-cell RNA sequencing]
TotalSeq™-C0301 anti-mouse Hashtag 1 Antibody Biolegend 155861
TotalSeq™-C0302 anti-mouse Hashtag 2 Antibody Biolegend 155863
TotalSeq™-C0307 anti-mouse Hashtag 7 Antibody Biolegend 155873
TotalSeq™-C0308 anti-mouse Hashtag 8 Antibody Biolegend 155875

[ChIP-seq]
rabbit anti-H3K4me3 Active Motif 39159

Validation

All antibodies were purchased and only used, according to the manufacturer's instructions. Validation statements are provided on the manufacture's website.

Animals and other research organisms

Policy information about studies involving animals; ARRIVE guidelines recommended for reporting animal research, and Sex and Gender in Research

Laboratory animals

Wild-type C57BL/6J mice were purchased from KBT Oriental (Breeder: Jackson Laboratory Japan). Il7r-Cre mice were provided by Professor Hans-Reimer Rodewald. GR-flox mice were provided by Professor Koichi Ikuta. All mice were housed in the specific pathogen-free animal facility at Kyushu University with a 12-hour light cycle.

Wild animals

Wild animals were not used in this study.

Reporting on sex

In experiments in which the adult offspring of treated mothers were given HDM nasally, the sexes were matched as closely as possible to the offspring of untreated mothers. In experiments in which lung ILC2 from adult offspring mice was sorted and cultured or transferred, adult offspring of OVA- or PBS- treated mothers were each matched to have an equal proportion of females and males. In transfer experiments, all sexes of recipient mice were female.

Field-collected samples

The study did not involved samples collected from the field.

Ethics oversight

All experimental procedures were reviewed and approved by the Kyushu University Animal Experiment Committee, and the care of the animals was in accordance with institutional guidelines.

Note that full information on the approval of the study protocol must also be provided in the manuscript.

Plants

Seed stocks	Not involved.
Novel plant genotypes	Not involved.
Authentication	Not involved.

ChIP-seq

Data deposition

☒ Confirm that both raw and final processed data have been deposited in a public database such as GEO.

☒ Confirm that you have deposited or provided access to graph files (e.g. BED files) for the called peaks.

Data access links<br>*May remain private before publication.*	Data have been deposited in the NCBI GEO databese under the accession number GSE262949.
Files in database submission	Raw fastq, processed bigwig and peaks.narrowPeak from adult offspring lung ILC2s of OVA- or PBS- treated mothers.
Genome browser session<br>(e.g. UCSC)	the Integrative Genomics Viewer (IGV) (version 2.17.0)

Methodology

Replicates	As the experiment was conducted once, no replicates were performed due to the experimental constraints.
Sequencing depth	Total: 4×10^8<br>Total bases: 30Gbp<br>Read length: 75+75bp.<br>Paired end
Antibodies	rabbit anti-H3K4me3 (Active Motif 39159)
Peak calling parameters	We called peaks using MACS2 (version 2.2.9.1) with the CallPeaks function to analyze ATACseq using Signac (version 1.12.0) on the RStudio platform (Version R 4.3.0). We did not perform peak calls on the ChIPseq data because we used the ChIPseq data for comparison with the peaks detected by ATACseq.
Data quality	We did not check the Data quality of the ChIPseq data as we used it to compare the ChIPseq data with the peaks detected by ATACseq.
Software	TrimGalore, Cutadapt, Bowtie 2, Sambamba, SAMtools, deepTools.

Flow Cytometry

Plots

Confirm that:

☐ The axis labels state the marker and fluorochrome used (e.g. CD4-FITC).

☒ The axis scales are clearly visible. Include numbers along axes only for bottom left plot of group (a 'group' is an analysis of identical markers).

☒ All plots are contour plots with outliers or pseudocolor plots.

☒ A numerical value for number of cells or percentage (with statistics) is provided.

Methodology

Sample preparation	BAL infiltrates were collected by 3 consecutive flushes of the airways with 1 mL of PBS. For the preparation of lung immune cells for flow cytometry analysis, lung tissue was digested in RPMI enzyme solution containing collagenase D (2 mg/ml, Roche, 11088882001) and DNase I (0.1 mg/ml, Roche, 10104159001) at 37°C for 60 min (for adults) or 40 min (for fetuses). Digestion was performed by changing the solution every 20 min. For the preparation of lung immune cells, fibroblast, and epithelial cells, lung tissues were digested in RPMI enzyme solution containing collagenase D (2 mg/ml), DNase I (0.1 mg/ml),

and Dispase II (0.2 mg/ml, Roche 04942078001) at 37°C for 40 min (for adults) or 20 min (for fetuses). Digestion was performed by changing the solution every 20 min. And then, lung tissues were digested in RPMI enzyme solution containing collagenase D (2 mg/ml), DNase I (0.1 mg/ml), Dispase II (0.2 mg/ml) and Liberase TH (0.01 mg/ml, Roche 5401151001) at 37° C for 20 min. The digested samples were filtered through 70 µm strainers and suspended in 36% Percoll (Cytiva, 17-0891-01) diluted with RPMI, 72% Percoll diluted with PBS was injected into the bottom and centrifuged at 800 × g for 20 min at 25°C. Cells were collected in the interlayer between 36% and 72% Percoll, washed with RPMI supplemented with FBS (2% v/v), and stained for flow cytometry. For BALF cells, the process of cell digestion and cell separation was not performed.

Instrument

Data were taken by LSRFortessa (BD Biosciences) or FACSMelody (BD Biosciences).

Software

Data were analyzed with FlowJo (version 10, BD Biosciences) and GraphPad Prism (Version 9).

Cell population abundance

Gating strategies was confirmed by backgating.

Gating strategy

Gating strategies for representative experiments were shown in Extended Data Fig. 7. All gating strategies captured cells by FSC-A/SSC-A area. FSC-A/FSC-H gates were used to exclude doublet cells.

For analysis:
Eosinophils were defined by CD45+CD11b+CD11c-Ly6G-SiglecF+.
Neutrophils were defined by CD45+CD11b+CD11c-Ly6G+SiglecF-.
B cells were defined by CD45+CD11b-CD11c-CD19+CD4-.
CD4+T cells were defined by CD45+CD11b-CD11c-CD19-CD4+.
Alveolar macrophages were defined by CD45+CD11b-CD11c+SiglecF+.
ILC2s were defined by CD45+lineage- (CD3e, CD4, CD8A, CD19, CD11b, CD11c, TER119, TCRɣδ) CD90.2+ST2+GATA3+.

For sorting of mouse fibroblasts, epithelial cells and ILC2s:
Fibroblasts were defined by CD45-EpCAM-CD31-CD140a+.
Epithelial cells were defined by CD45-EpCAM+CD31-.
In adult mouse lungs, ILC2s were defined by CD45+lineage- (CD3e, CD4, CD8A, CD19, CD11b, CD11c, TER119) CD90.2+ST2+.
In fetal mouse lungs, ILC2s were defined by CD45+lineage- (CD3e, CD4, CD8A, CD19, CD11b, CD11c, TER119) ST2+.

☒ Tick this box to confirm that a figure exemplifying the gating strategy is provided in the Supplementary Information.
